# Supplementary material for: Body coloration and mechanisms of colour production in Archelosauria: the case of deirocheline turtles
Source: R Soc Open Sci. 2019 Jul 24;6(7):190319. doi: 10.1098/rsos.190319 (PMC6689573; doi:10.1098/rsos.190319)
Supplement: Supplementary materials from: Body coloration and mechanisms of colour production in Archelosauria: The case of deirocheline turtles [file rsos190319supp1.pdf]

Body coloration and mechanisms of colour production in Archelosauria: The case of deirocheline turtles

Jindřich Brejcha, José Vicente Bataller, Zuzana Bosáková, Jan Geryk, Martina Havlíková, Karel Kleisner, Petr Maršík, Enrique Font

SUPPLEMENTARY MATERIALS

Table S1 Elution gradient of UPLC separation.

Time [min]	0	12	30	33	43	45	47	57
**A** (MeCN) [%]	20	100	5	0	0	100	20	20
**B** (MeOH/H2O, 1:1 v/v/) [%]	80	0	0	0	0	0	80	80
**C** (TBME/MeCN/MeOH, 86/86/8 v/v/v) [%]	0	0	95	100	100	0	0	0

MeCN – Acetonitrile; MeOH – Methanol; TBME – tert-Butyl Methyl Ether

Table S2 HRAM Q-TOF MS conditions.

	ESI ionization source	APCI ionization source
Capillary voltage	4500 V	4000 V
End Plate Offset	-500 V	-500 V
Charging Voltage	2000 V	2000 V
Nebulizer	0.3 Bar	2.5 Bar
Dry Heater	250 °C	250 °C
Dry Gas	4.0 l/min	4.0 l/min
Scan range	60-1500 m/z	60-1500 m/z
Corona curr	-	4000 nA
APCI Heater	-	450 °C

Table S3 SRM conditions used for LC-MS/MS determination of the pterins and riboflavin.

Compound	Molecular weight	Precursor ion	Product ion	Fragmentor (V)	Collision energy (V)
L-Sepiapterin	237.2	238.2	192.1	120	15
Pterin	163.1	164.1	119.1	100	20
Pterin-6-carboxylic acid	209.2	208.1	162.1	100	15
6-Biopterin*	237.1	238.1	178.1	115	17
Isoxanthopterin*	179.1	180.1	135.1	125	20
Xanthopterin*	179.1	180.1	135.1	125	20
Leucopterin*	195.1	196.1	140.1	120	16
Erythropterin*	265.1	266.1	220.1	110	8
D-Neopterin*	253.2	254.2	206.2	115	14
Drosopterin +	368.4	369.4	230.2	125	30
Riboflavin +	376.4	377.4	243.3	135	30

*For pterins marked by asterisk the MS/MS conditions was adopted from Kozlík et al. (2013)[1]

+ For drosopterin and riboflavin the MS/MS conditions were adopted from Andrade et al. (2019)[2]

[1] Kozlík P, Krajíček J, Kalíková K, Tesařová E, Čabala R, Exnerová A, Štys P, Bosáková Z. 2013 Hydrophilic interaction liquid chromatography with tandem mass spectrometric detection applied for analysis of pteridines in two Graphosoma species (Insecta: Heteroptera). ***J. Chromatogr. B 930,*** 82–89

[2] Andrade P et al. 2019 Regulatory changes in pterin and carotenoid genes underlie balanced color polymorphisms in the wall lizard. ***PNAS, 116, 5633-5642.***

Figure S1 Results of analyses of carotenoids. A) UPLC chromatograms (at 472nm) of mixture of carotenoid standards. B) UPLC chromatograms (at 472nm) resulting from carotenoid analyses of differen turtle skin samples with retention times of predominant carotenoids. Red chromatogram represents red postorbital region of *Trachemys scripta elegans*, all other regions are by different colors. Absorbance spectra C) of standard of lutein, D) of standard of zeaxanthin, E) of standard of cantaxanthin, F) of standard of astaxanthin. G-J absorbance spectra of the four main carotenoid types found in the skin of turtles (compare the wavelenght of the highest peak in nm, and the retention time in min. to absorbance spectra of the carotenoid standards).

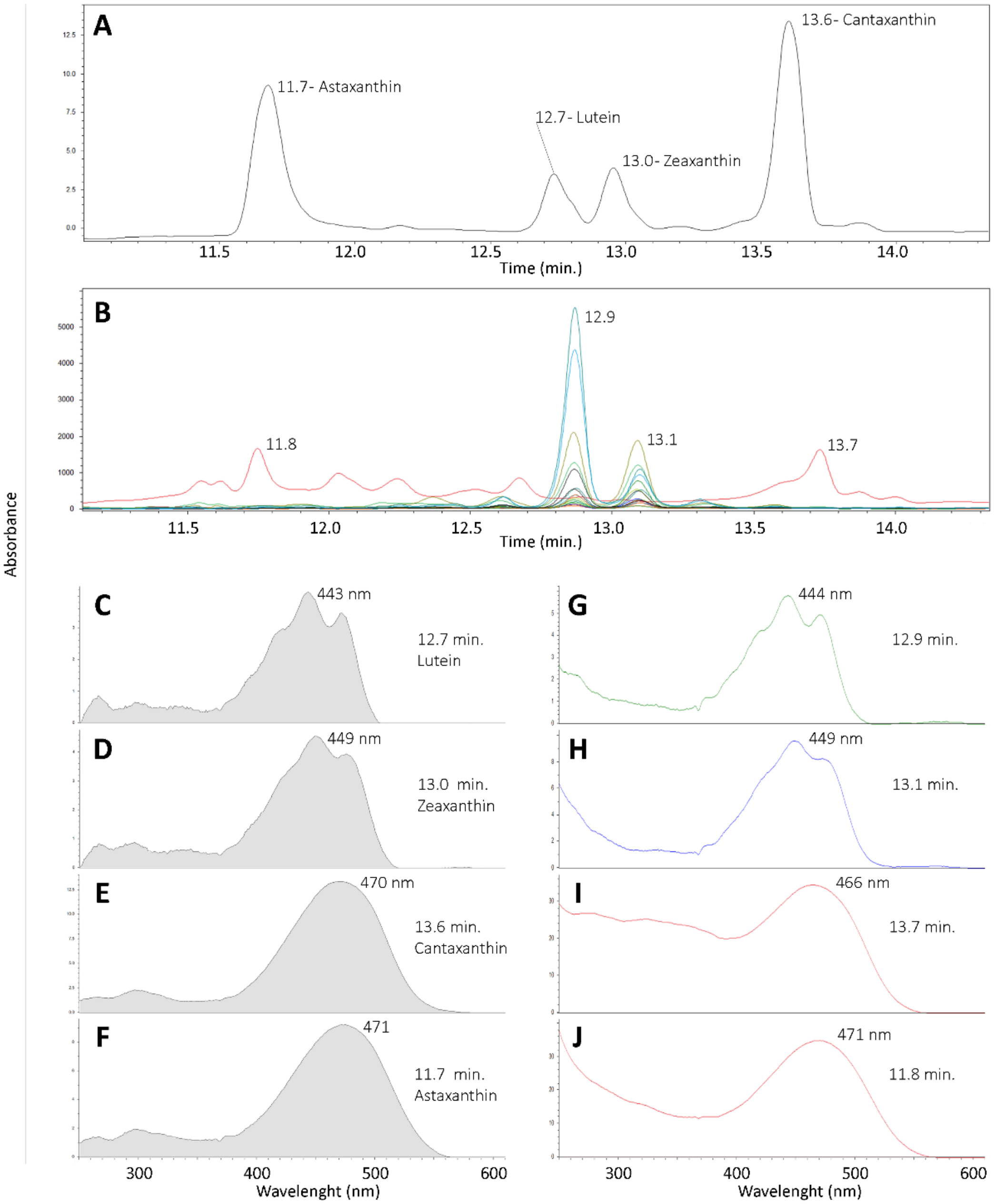

Figure S2 Chromatograms of standards of pterins and riboflavin. A) TIC chromatogram of the mixture of studied pterins (with exception of drosopterin) and riboflavin (c = 0.01 mg/ml),1-riboflavin, 2- L-sepiapterin, 3-pterin, 4-isoxanthopterin, 5-biopterin, 6-xantopterin, 7-leucopterin, 8-erytropterin, 9-D-neopterin, 10-pterin-6-COOH. B) SRM chromatograms of the all studied pteridine derivatives measured in the mixture (c = 0.01 mg/ml). C) SRM chromatogram of drosopterin extract.

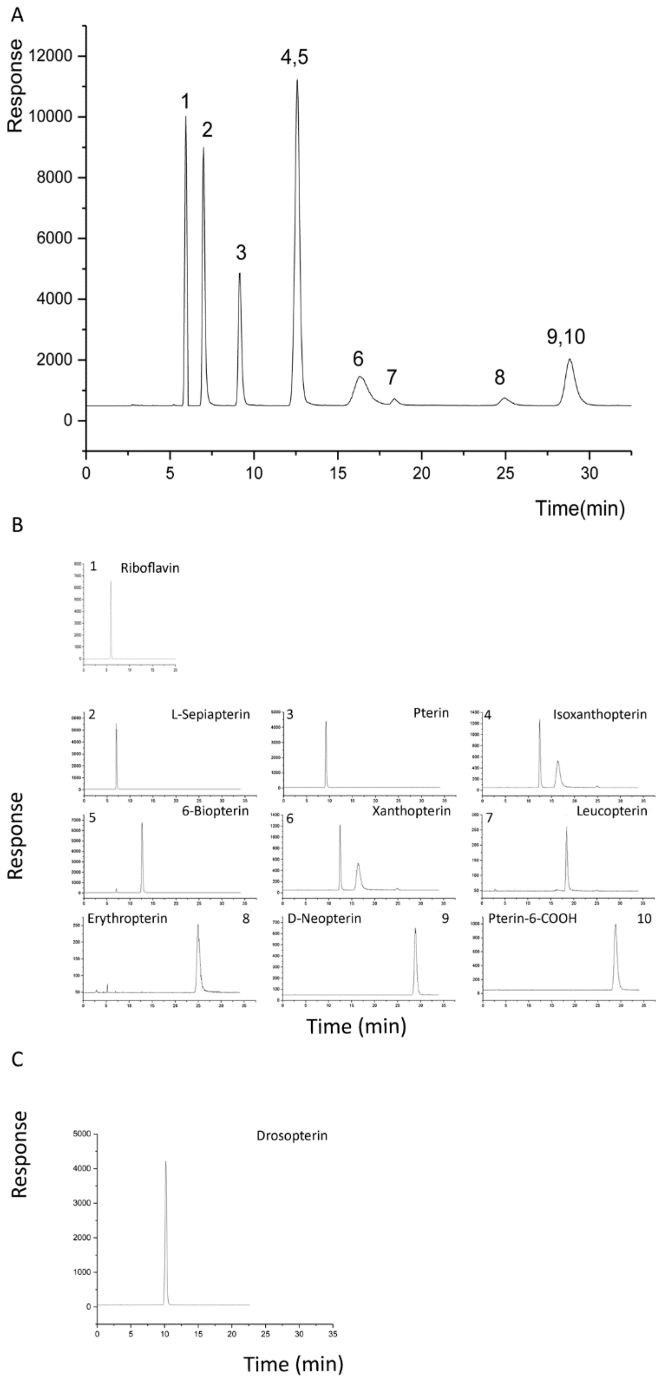

Figure S3 Distributions of reflective platelets' orientation and width. A) orientation of reflective platelets in main median chin yellow stripe (CBC) of *Pseudemys concinna*. B) orientation of reflective platelets in postorbital marking of *P. concinna*. C) orientation of reflective platelets in CBC region of *Trachemys scripta*. D) orientation of reflective platelets in main bright stripe of the fore limb (FLBS) of *T. scripta*. E) reflective platelets width of CBC region of *P. concinna*. F) reflective platelets width of PM region of *P. concinna*. G) reflective platelets width of CBC region of *T. scripta*. H) reflective paltelets width of FLBS region of *T. scripta*.

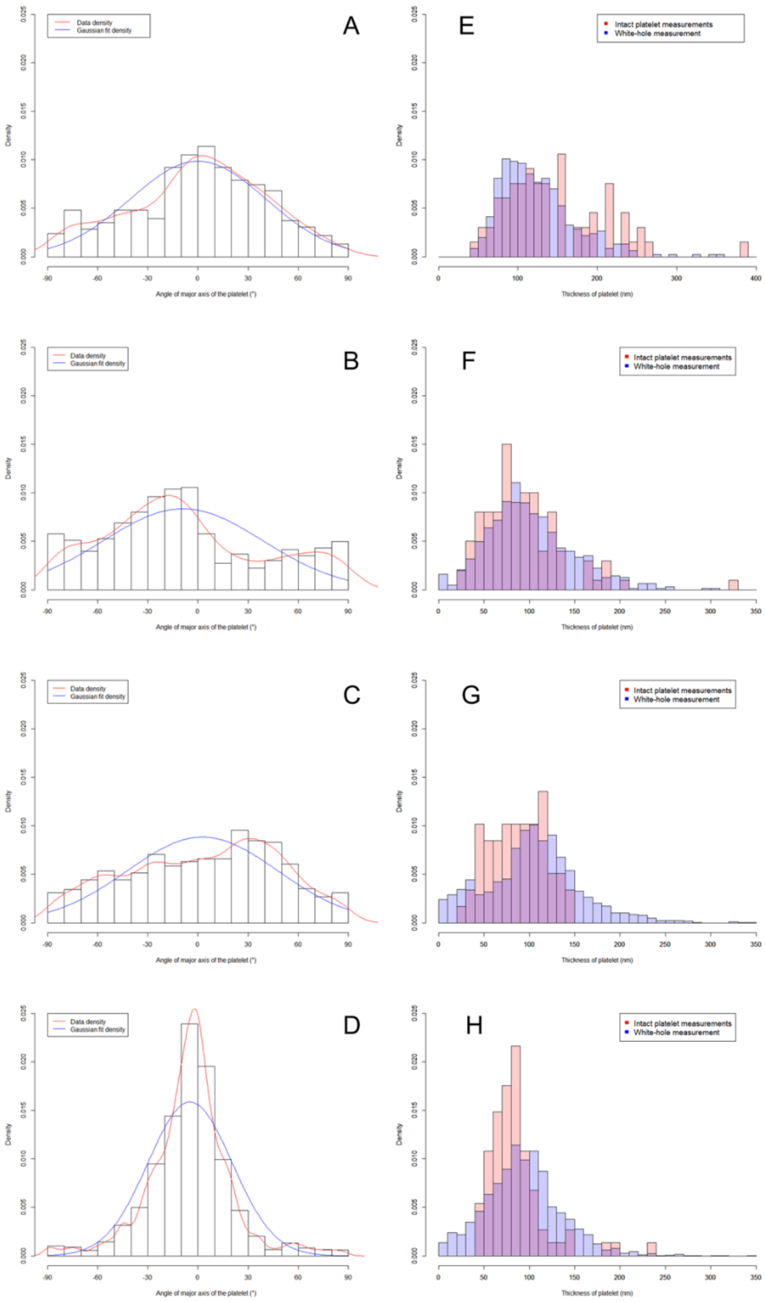

Figure S4 Variation in 2D Fourier power spectra of spatial distribution of dermal collagen fibers of freshwater turtles. A) Postorbital marking (PM) of the *Trachemys scripta*. B) Main median chin yellow stripe (CBC) of *T. scripta*. C) Main bright stripe forelimb stripe of *T. scripta*. D) PM region of *Pseudemys concinna*. E) CBC region of *P. concinna*. F) FLBS region of *P. concinna*.

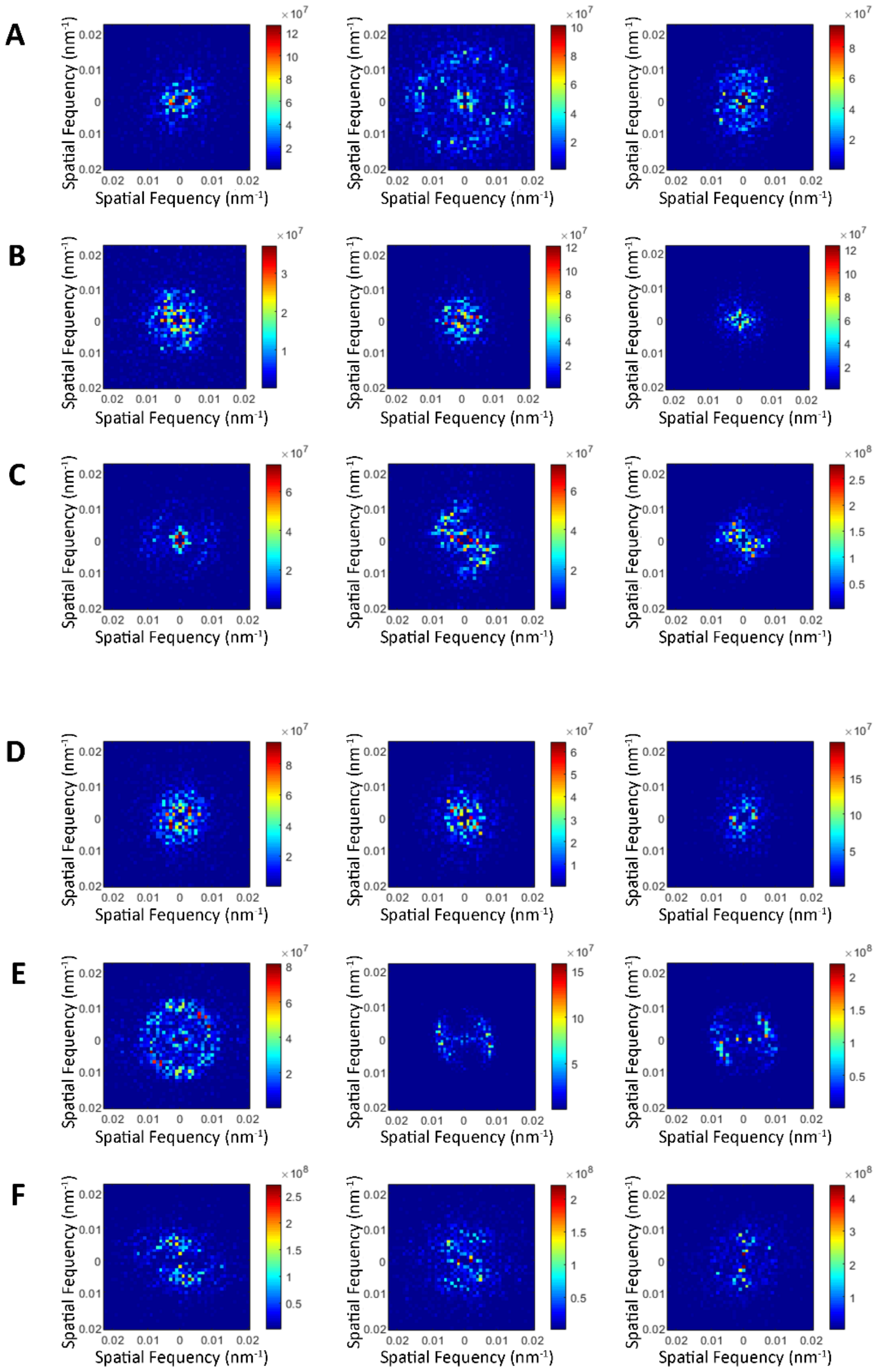

Figure S5 Results of Fourier analyses of collagen fibers in yellow zygomatic patch (ZP) of *Trachemys scripta scripta*. A) Radial means of Fourier power. B) Measured reflectance spectra (solid line) compared to Fourier predicted reflectivity (dashed line).

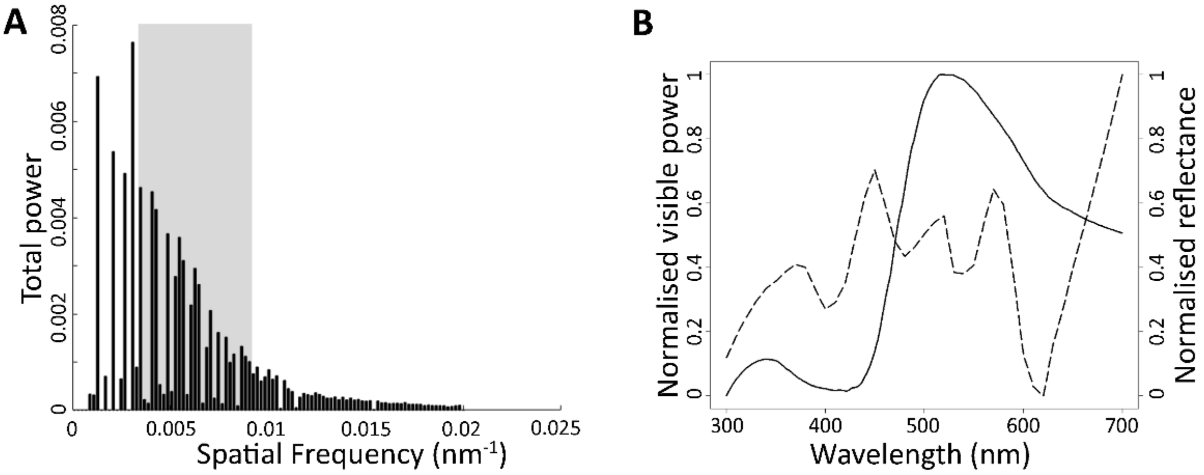

Figure S6 Results of analyses of pterins. A) Example of SRM chromatograms of the red postorbital region of *Trachemys scripta elegans*. B) Example of SRM chromatograms of the yellow regions of *Trachemys scripta*. C) Example of SRM chromatograms of the yellow regions of *Pseudemys concinna*.

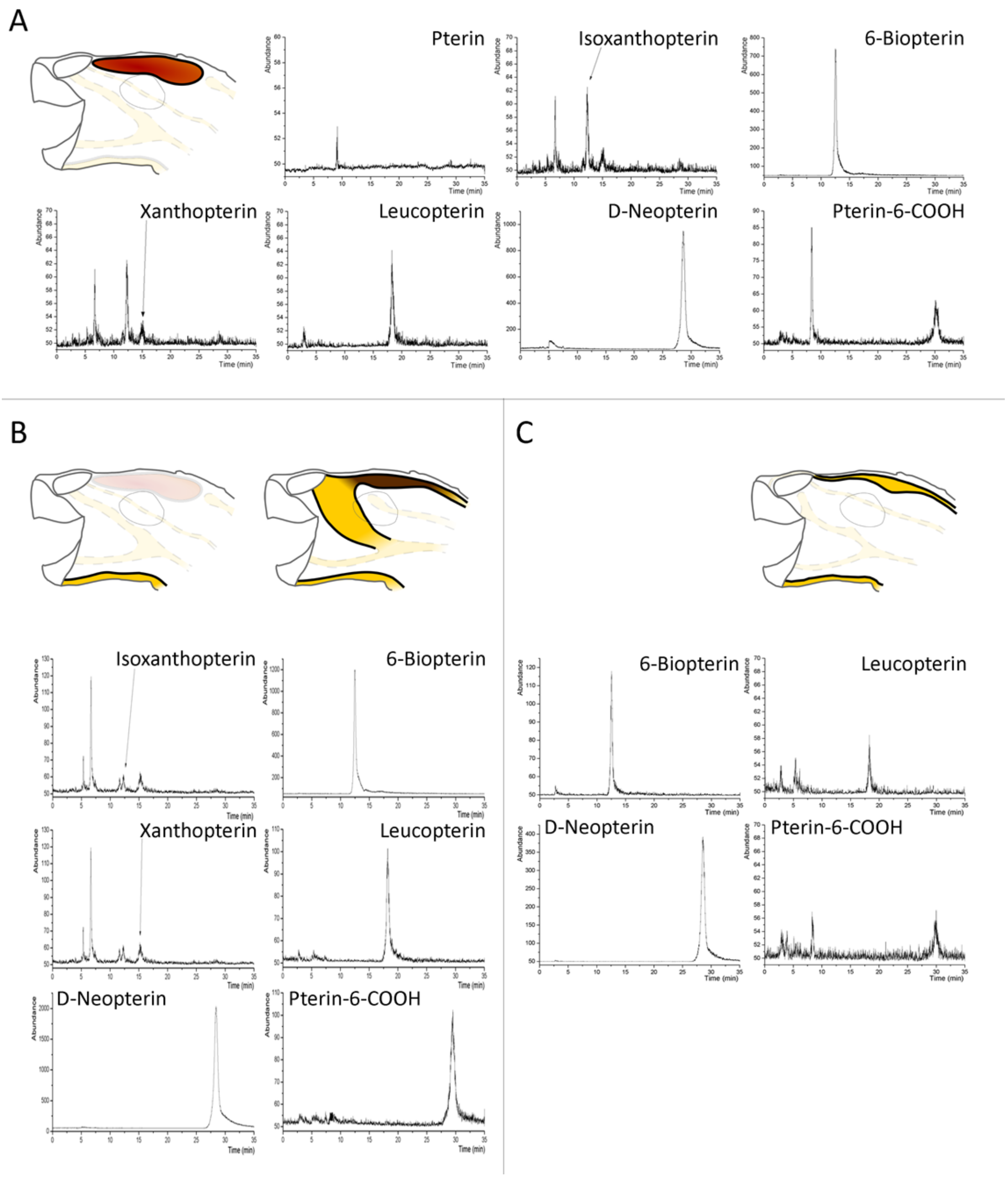

Figure S7 Differences in colour between sexes of *Pseudemys concinna* and *Trachemys scripta elegans*. A) Biplot representing results of RDA of summary variables derived from reflectance spectra of *Pseudemys concinna* and *Trachemys scripta elegans*. First axis (RDA1) is constrained by species (explains 18 % of total variance). Second axis (RDA2) is constrained by sex (explains 4 % of total variance). Dark grey hull denotes individuals of *P. concinna*, light grey hull denotes individuals of *T. scripta*, pink hull denotes females, blue hull denotes males. Summary variables are denoted by names, but when spacing would not allow to read names of variables clearly, variables are denoted by crosses. Description of summary variables are in the main text of the article. B) Biplot representing results of RDA of summary variables derived from reflectance spectra of *T. s. elegans*. First axis (RDA1) is constrained by sex (explains 5,5 % of total variance). First residual axis PC1 explains 21 % of total variance. Pink hull denotes females, blue hull denotes males. C) Results of RDA of summary variables derived from reflectance spectra of *P. concinna* with first axis constrained by sex were not significant.

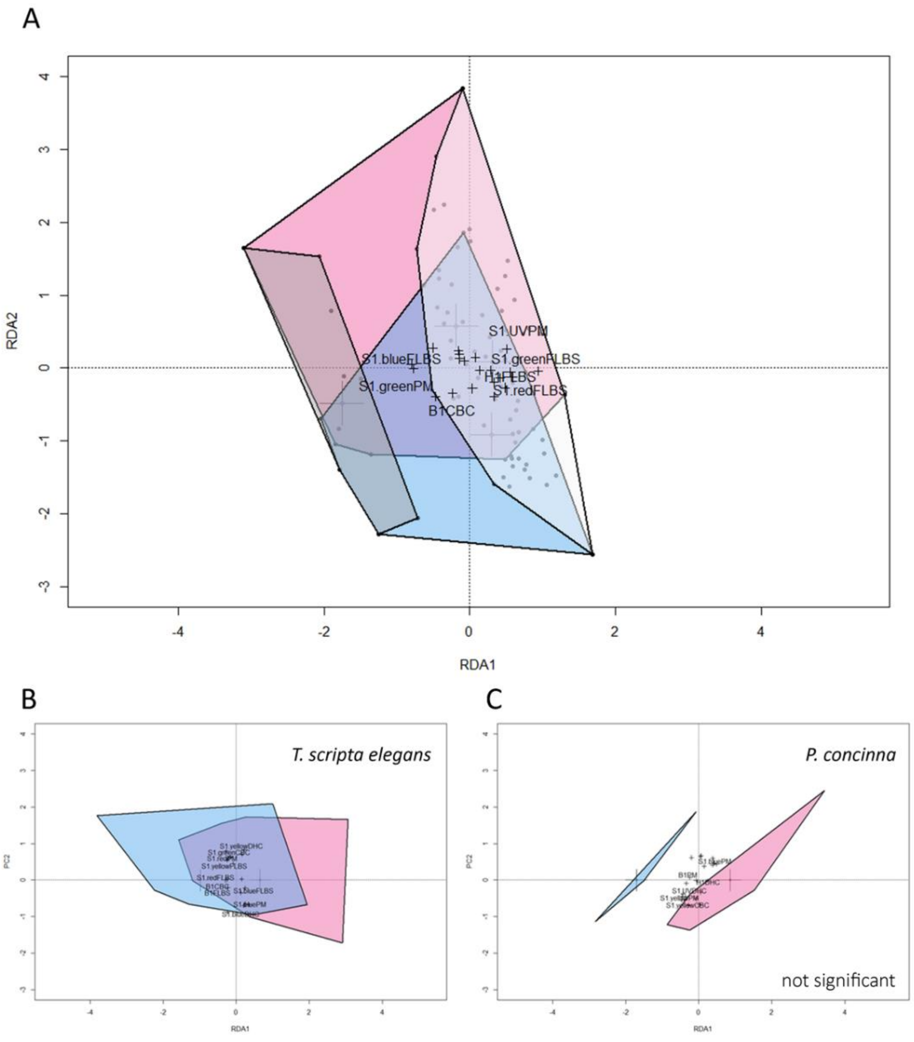

Table S4 Results of ANOVAs comparing summary variables from reflectance spectra between sexes

Species	Summary variable	F	p value	adjusted p
Trachemys scripta elegans				
	B1CBC	13.22	0.0005	0.0141
	S1.UVCBC	1.86	0.1767	1.0000
	S1.blueCBC	2.77	0.1009	1.0000
	S1.greenCBC	1.61	0.2089	1.0000
	S1.yellowCBC	3.42	0.0690	1.0000
	S1.redCBC	2.77	0.1011	1.0000
	H1CBC	0.67	0.4167	1.0000
	B1DHC	1.11	0.2949	1.0000
	S1.UVDHC	2.73	0.1035	1.0000
	S1.blueDHC	0.67	0.4158	1.0000
	S1.greenDHC	1.03	0.3149	1.0000
	S1.yellowDHC	0.92	0.3413	1.0000
	S1.redDHC	2.16	0.1466	1.0000
	H1DHC	1.13	0.2926	1.0000
	B1FLBS	12.98	0.0006	0.0151
	S1.UVFLBS	2.65	0.1084	1.0000
	S1.blueFLBS	10.89	0.0016	0.0375
	S1.greenFLBS	0.30	0.5850	1.0000
	S1.yellowFLBS	4.71	0.0336	0.6714
	S1.redFLBS	17.22	0.0001	0.0026
	H1FLBS	3.87	0.0534	1.0000
	B1PM	2.41	0.1256	1.0000
	S1.UVPM	5.32	0.0243	0.5096
	S1.bluePM	6.83	0.0111	0.2464
	S1.greenPM	0.96	0.3297	1.0000
	S1.yellowPM	1.92	0.1706	1.0000
	S1.redPM	6.90	0.0107	0.2464
Pseudemys concinna				
	B1CBC	1.15	0.3086	1.0000
	S1.UVCBC	2.94	0.1169	1.0000
	S1.blueCBC	0.03	0.8741	1.0000
	S1.greenCBC	1.49	0.2495	1.0000
	S1.yellowCBC	1.69	0.2224	1.0000
	S1.redCBC	1.52	0.2458	1.0000
	H1CBC	0.05	0.8238	1.0000
	B1DHC	0.69	0.4266	1.0000
	S1.UVDHC	1.09	0.3209	1.0000
	S1.blueDHC	0.00	0.9475	1.0000
	S1.greenDHC	1.04	0.3322	1.0000
	S1.yellowDHC	0.38	0.5540	1.0000
	S1.redDHC	0.08	0.7879	1.0000
	H1DHC	1.20	0.2992	1.0000
	B1FLBS	0.08	0.7881	1.0000
	S1.UVFLBS	2.87	0.1211	1.0000
	S1.blueFLBS	3.69	0.0836	1.0000
	S1.greenFLBS	3.55	0.0890	1.0000
	S1.yellowFLBS	5.39	0.0426	1.0000
	S1.redFLBS	5.19	0.0459	1.0000
	H1FLBS	0.09	0.7726	1.0000
	B1PM	1.67	0.2259	1.0000
	S1.UVPM	5.87	0.0358	0.9315
	S1.bluePM	4.88	0.0516	1.0000
	S1.greenPM	2.67	0.1336	1.0000
	S1.yellowPM	8.44	0.0157	0.4239
	S1.redPM	4.27	0.0657	1.0000
